# Confirmed Cases of Ophidiomycosis in Museum Specimens from as Early as 1945, United States

**DOI:** 10.3201/eid2707.204864

**Published:** 2021-07

**Authors:** Jeffrey M. Lorch, Steven J. Price, Julia S. Lankton, Andrea N. Drayer

**Affiliations:** US Geological Survey National Wildlife Health Center, Madison, Wisconsin, USA (J.M. Lorch, J.S. Lankton);; University of Kentucky, Lexington, Kentucky, USA (S.J. Price, A.N. Drayer)

**Keywords:** emerging infectious disease, museums, mycoses, reptiles, snakes, fungi, United States

## Abstract

Ophidiomycosis represents a conservation threat to wild snake populations. The disease was reported in North America early in the 21st century, but the history of ophidiomycosis has not been investigated. We examined museum specimens and confirmed cases of ophidiomycosis >50 years before the disease’s reported emergence.

Emerging fungal pathogens of wildlife are recognized as major threats to global biodiversity, causing population declines and extinction events in a variety of host species (*1*). *Ophidiomyces ophidiicola*, the causative agent of ophidiomycosis, is one such pathogen recognized as a conservation threat to wild snakes (*2*). The disease first gained attention in 2008 when fatal infections emerged in eastern massasauga rattlesnakes (*Sistrurus catenatus*) in Illinois, USA (*3*), and has since been documented throughout North America and Europe (*2*,*4*). The earliest retrospective detection of *O. ophidiicola* in snakes was from 2000 (*5*). We report the earliest known confirmed cases of ophidiomycosis in free-living snakes in the United States, dating back to 1945.

We investigated the historical occurrence of ophidiomycosis in snakes in the United States by examining specimens preserved in formalin or ethanol at the University of Wisconsin Zoological Museum (UWZM; Madison, WI, USA) and Morehead State University Museum Collection (Morehead, KY, USA). We visually examined 524 specimens representing 30 snake species from 19 states in the eastern United States collected during 1900–2012 (Appendix 1). To reduce risk for cross-contamination, we first examined snakes for clinical signs of ophidiomycosis within the glass jars in which they were stored. When specimens were removed from the jars for sampling, new gloves were worn to handle each snake. We observed clinical signs consistent with ophidiomycosis (Figure) in 47 (9.0%) snakes (*6*). These specimens represented 12 species from 7 states with collection dates ranging from 1929 to 1983 (Appendix 1).

**Figure:**
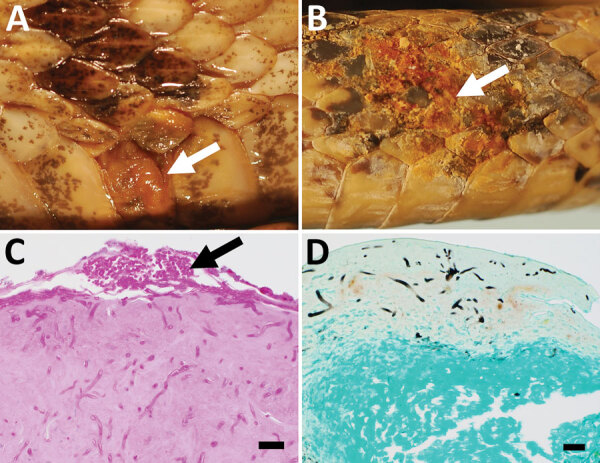
Gross and histologic lesions in museum snake specimens with confirmed ophidiomycosis, United States. A, B) *Crotalus horridus* (A; University of Wisconsin Zoology Museum [UWZH] accession no. 22773) and *Cemophora coccinea* (B; UWZH accession no. 13822) specimens with thickened necrotic scales (arrows). C, D) Histologic sections of lesioned skin from the same *C. horridus* (C; UWZH accession no. 22773) and *C. coccinea* (D; UWZH accession no. 13822) specimens showing arthroconidia (arrow) and intralesional fungal hyphae consistent with *Ophidiomyces ophidiicola* infection. Scale bars indicate 20 µm.

Clinical signs of ophidiomycosis are not pathognomonic, and a confirmed diagnosis requires compatible histopathologic lesions and the detection of *O. ophidiicola* (*6*). Because these confirmatory steps involve destructive sampling of museum material, we selected a subset of snakes (n = 12) for these analyses. We targeted specimens with large (>0.5 cm^2^) or multiple skin lesions from distant geographic areas and collected >25 years before the reported 2008 emergence of ophidiomycosis (*3*) (Table). From selected snakes, we excised and formalin-fixed portions of lesioned skin, routinely processed them for light microscopy, and stained with periodic acid-Schiff and Grocott methenamine silver methods. We also collected small pieces of lesioned skin (≈4 mm^2^) for PCR-based detection of *O. ophidiicola*. We extracted DNA from dehydrated tissue by using the Gentra Puregene Tissue Kit (QIAGEN, https://www.qiagen.com); we used 10 µL of the kit-provided proteinase K per sample. Negative controls consisted of blank extractions. For PCR, we used existing primers that specifically target the internal transcribed spacer region (ITS) of *O. ophidiicola* (*6*) and a newly designed PCR assay that targets mitochondrial NADH dehydrogenase subunit 1 (nad1) (Appendix 2). We targeted these 2 loci, which exist at high copy numbers in the genome, because amplifiable DNA was expected to be at low abundance in the preserved specimens. We cloned and sequenced PCR amplicons of the appropriate size to confirm the presence of *O. ophidiicola*. We conducted tissue collection, DNA extraction, and PCR under strict protocols (e.g., unidirectional workflow and regular decontamination of work surfaces and equipment) to prevent contamination of samples.

**Table T1:** Museum snake specimens with clinical signs of ophidiomycosis that were subjected to histopathologic examination and PCR specific for *Ophidiomyces ophidiicola*, United States*

Snake species	Museum accession no.	Museum collection	State collected	Date collected	Ophidiomycosis histopathology	PCR result†	Ophidiomycosis diagnosis‡
*Crotalus horridus*	UWZH 22773	UWZM	WI	1958 Aug	Positive	Positive	Confirmed
*C. horridus*	UWZH 23927	UWZM	TN	1973 Apr 13	Positive	Negative	Apparent
*C. horridus*	UWZH 23930	UWZM	TN	1973 Apr 13	Positive	Negative	Apparent
*Cemophora coccinea*	UWZH 13833	UWZM	FL	1945	Positive	Positive	Confirmed
*Lampropeltis triangulum*	UWZH 22583	UWZM	WI	1982 Apr 25	Positive	Negative	Apparent
*Pantherophis spiloides*	UWZH 23931	UWZM	TN	1973 Apr 13	Positive	Positive	Confirmed
*Agkistrodon contortrix*	582	MSUMC	KY	1979 Oct 29	Equivocal§	Negative	Possible
*Coluber constrictor*	603	MSUMC	KY	1980 May 6	Positive	Negative	Apparent
*C. constrictor*	632	MSUMC	KY	1980 May 16	Negative	Negative	Possible
*Regina septemvittata*	496	MSUMC	KY	1979 May 30	Equivocal	Negative	Possible
*R. septemvittata*	511	MSUMC	KY	1979 Jun 2	Equivocal	Negative	Possible
*R. septemvittata*	634	MSUMC	KY	1980 May 18	Equivocal	Negative	Possible

*MSUMC, Morehead State University Museum Collection; *Oo*, *Ophidiomyces ophidiicola*; UWZM, University of Wisconsin Zoology Museum. †Samples are listed as positive if >1 PCR assay targeting the internal transcribed spacer region or mitochondrial NADH dehydrogenase subunit 1 gene was positive. See Appendix 1 for assay-specific results. ‡Based on Baker et al. (*5*). §Equivocal indicates some histologic features consistent with ophidiomycosis were present but >1 diagnostic features were not observed (Appendix 1)[Table T1].

Of the 12 snakes subjected to histopathological analyses, 7 (58.3%) had microscopic lesions with intralesional fungi consistent with ophidiomycosis (*6*) (Table; Figure). We detected DNA from *O. ophidiicola* in 3 (50%) of the 6 specimens from UWZM that had been stored in 70% ethanol (Table). We did not detect DNA of *O. ophidiicola* in snakes from the Morehead State University Museum Collection (n = 6), likely because these specimens were stored long-term in formalin, which is known to affect the recovery of amplifiable nucleic acid. These results highlight the importance of targeting specimens stored in ethanol rather than formalin for molecular-based detection of pathogens in archival material.

We amplified the ITS target from 2 of the 3 specimens and nad1 target from all 3 specimens; these sequences were 100% identical to existing *O. ophidiicola* sequences in GenBank. The 3 additional specimens from UWZM were strongly suspected to represent cases of ophidiomycosis on the basis of the presence of arthroconidia in histologic sections of lesioned skin (*6*); however, fungal DNA from these specimens may not have been suitable for PCR amplification. Negative controls performed as expected. The 3 PCR-positive specimens met the diagnostic criteria for confirmed cases of ophidiomycosis (*6*); they were collected in Florida in 1945, Wisconsin in 1958, and Tennessee in 1973 (Table). These cases predate the earliest previously known detection of *O. ophidiicola* in free-living snakes in North America by as much as 55 years (*5*).

Museum specimens can provide crucial insights into the history of emerging infectious diseases. Preserved animal specimens have been used to trace the origin and spread of other fungal pathogens, such as *Pseudogymnoascus destructans* (white-nose syndrome in bats) and *Batrachochytrium* spp. (chytridiomycosis in amphibians) (*8*–*10*). By using a similar approach, we demonstrate that ophidiomycosis was circulating in the eastern United States for decades before its recognition as an emerging disease. Future work focusing on how such factors as climate change, environmental disturbance, and underlying health of snake populations influence ophidiomycosis dynamics might reveal the mechanism by which ophidiomycosis is emerging (*2*).

Appendix 1 Additional data about confirmed cases of ophidiomycosis in museum specimens from as early as 1945, United States

Appendix 2 Additional information about confirmed cases of ophidiomycosis in museum specimens from as early as 1945, United States
