# Supplementary material for: Confirmed Cases of Ophidiomycosis in Museum Specimens from as Early as 1945, United States
Source: Emerg Infect Dis. 2021 Jul;27(7):1986–9. doi: 10.3201/eid2707.204864 (PMC8237876; doi:10.3201/eid2707.204864)
Supplement: Appendix 2 — Additional information about confirmed cases of ophidiomycosis in museum specimens from as early as 1945, United States [file 20-4864-Techapp-s2.pdf]

Article DOI: https://doi.org/10.3201/eid2707.204864

Confirmed Cases of Ophidiomycosis in Museum Specimens from as Early as 1945, United States

Appendix 2

PCR-Based Detection of *Ophidiomyces ophidiicola* in Museum Specimens

An 82-nt portion of the internal transcribed spacer region of *Ophidiomyces ophidiicola* (*Oo*) was targeted for amplification by using the previously described (*1*) primers Oo-rt-ITS-F (5′ – GAGTGTATGGGAATCTGTTTC – 3′) and Oo-rt-ITS-R (5′ – GGTCAAACCGGAAAGAATG – 3′) under the following cycling conditions: 94°C for 5 minutes; 48 cycles of 94°C for 30 seconds, 58.8°C for 30 seconds, 68°C for 30 seconds; and a final extension at 68°C for 5 minutes.

Formalin fixation of tissues is known to reduce or eliminate amplifiable nucleic acids, and successful amplification is dependent on the target sequence being intact. For this reason, we designed a second PCR assay that targeted a small fragment of the NADH dehydrogenase subunit 1 (nad1) present on the mitochondrial genome of *Oo*. Because a large quantity of mitochondria can be present within a single fungal cell, nad1 potentially exists at a higher copy number than the internal transcribed spacer region and therefore may be more likely to amplify in samples for which little amplifiable DNA remains. Primers Oo-nad1-F (5′ – ACTTGATTGTTTCTCTAGTC – 3′) and Oo-nad1-R (5′ – AGGGAAAGAAGCTCTAAC – 3′) were designed to amplify an 85-nt portion of nad1 that exhibits a high degree of interspecific variability for ascomycete fungi. Cycling conditions for the PCR were as follows: 94°C for 5 min; 48 cycles of 94°C for 30 sec, 50°C for 30 sec, 68°C for 30 sec; and a final extension at 68°C for 5 min. In a screening test, the assay was shown to amplify the nad1 region of three *Oo* isolates tested: strains UAMH 10296 (UAMH Centre for Global Microfungal Biodiversity Culture Collection), ATCC MYA-4974 (American Type Culture Collection), NWHC 23913–1 (US Geological Survey National Wildlife Health Center Culture Collection); GenBank accession

numbers MW358097-MW358099. However, the assay did not amplify DNA from 19 closely related fungi in the order Onygenales (*Arthroderma quadrifidum* strain UAMH 2941, *Chrysosporium indicum* strain UAMH 10212, *Nannizziopsis arthrosporoides* strain CBS 133988 [Centraalbureau voor Schimmelcultures], *N. chlamydospora* strain CBS 133985, *N. crocodili* strain UAMH 9666, *N. dermatitidis* strain UAMH 7583, *N. draconii* strain CBS 133987, *N. guarroi* strain CBS 124553, *N. hominis* strain UAMH 7859, *N. infrequens* strain UAMH 10417, *N. obscura* strain UAMH 5875, *N. pluriseptata* strain CBS 133989, *N. vriesii* strain UAMH 3527, *Paranannizziopsis californiense* strain UAMH 10693, *Paranannizziopsis crustacea* strain UAMH 10199, *Paranannizziopsis longisporum* strain CBS 133990, *Pseudoamauroascus australiensis* strain UAMH 8392, *Trichophyton terrestre* strain UAMH 657, and *Uncinocarpus reesii* strain UAMH 3880) or other fungi commonly found on the skin of snakes (*2*; *Purpureocillium lilacinum* strain NWHC24022–1, *Bionectria* sp. strains NWHC26452–1 and NWHC26464–4), indicating that the assay was specific to *Oo*.

References

1. Bohuski E, Lorch JM, Griffin KM, Blehert DS. TaqMan real-time polymerase chain reaction for detection of *Ophidiomyces ophiodiicola*, the fungus associated with snake fungal disease. BMC Vet Res. 2015;11:95. PubMed https://doi.org/10.1186/s12917-015-0407-8
